# ICU tracheotomies in patients with COVID-19: a lesson learned for future viral pandemic

**DOI:** 10.1007/s00405-022-07360-4

**Published:** 2022-05-12

**Authors:** Gradys Agata, Szrama Jakub, Nogal Piotr, Wierzbicka Małgorzata, Kusza Krzysztof

**Affiliations:** 1 Department of Anesthesiology, Intensive Therapy and Pain Management, Poznan University of Medical Sciences, Poznan, Poland; 2 Department of Otolaryngology and Oncological Laryngology, Poznan University of Medical Sciences, Poznan, Poland; 3 Department of Anesthesiology, Intensive Care and Pain Management, Poznan University of Medical Sciences, Poznan, Poland

**Keywords:** COVID-19, Open surgical tracheotomy, Percutaneous tracheotomy, Tracheotomy

## Abstract

**Introduction:**

The coronavirus SARS-CoV-2 pandemic has resulted in a large number of patients requiring intubation and prolonged mechanical ventilation. The current knowledge on the tracheotomies regarding the time form intubation, method and ventilatory parameters optimal for their performance in the mechanically ventilated patients with COVID ARDS are scarce; thus, the aim of this study is to present new data regarding their safety, adverse events and timing.

**Materials and methods:**

This retrospective observational study is based on the data of 66 critically ill COVID patients including demographic data, timing and technique of tracheotomy, ventilatory parameters in the time of procedure, as well as complication and survival rate.

**Results:**

A number of 66 patients with COVID-related pneumonia were included in the study, among whom 32 were tracheotomized—25 patients underwent an early tracheotomy and 7 patients had late tracheotomy. The median duration of mechanical ventilation before the tracheotomy in the early group was 8 days (IQR 6–10) compared to 11 days (IQR 11–12.5.) *p* < 0.001) in late group. Risk of death in tracheotomy patients was significantly growing with growing level of PEEP and FiO2 at the moment of decision on tracheotomy, OR = 1.91 CI_95_ (1.23;3.57); *p* = 0.014 and OR = 1.18 CI_95_(1.03;1.43); *p* = 0.048, respectively.

**Conclusion:**

Early percutaneous tracheotomy is safe (both in terms of risk of viral transmission and complication rate) and feasible in COVID-19 patients. Stability of gas exchange, and ventilatory parameters are the main prognostic factors of the outcome.

## Introduction

The coronavirus SARS-CoV-2 pandemic causing acute respiratory failure has resulted in a large number of patients requiring intubation and prolonged mechanical ventilation. The intensive care guidelines give a lot of information about mechanical ventilation in the ARDS patients, including lung protective ventilation, prone position and use of muscle relaxants [1, 2]. Despite the tremendous number of studies analysing the very detailed aspect of mechanical ventilation, the data on the tracheotomies in the ARDS patients are scarce. In comparison to the orotracheal tube, the tracheotomy tube can prevent complications related to the oropharyngeal and laryngeal lesions, such as postintubation subglottic stenosis [3], improves patient comfort, allows a decrease in doses of sedative drugs, facilitates weaning from mechanical ventilation and potentially reduces the rate of ventilator associated pneumonia [4, 5]. There are no guidelines on the timing, method and ventilatory parameters optimal for the performance of the tracheotomy. The UK and North America recommendations suggest delaying the tracheotomy until 14 days of mechanical ventilation to allow better prognosis of the outcome and to reduce the viral load of the patients [6–9]. On the other hand, the French group favours an early tracheotomy to facilitate weaning and transfer patients to a ventilatory weaning unit making free ICU beds for new patients [10].

The aim of the study was to describe the clinical data and outcome of critically ill COVID patients undergoing tracheotomy and to evaluate its timing, safety and adverse events, as well as to compare mortality, duration of mechanical ventilation, length of ICU stay and VAP occurrence between tracheotomized and non-tracheotomized patients, and between an early and late tracheotomy group.

## Materials and methods

This retrospective observational study was performed in an Intensive Care Unit in Poznan University Hospital in Poland. The tracheotomies were categorized as early, when performed before the 10th day of mechanical ventilation, and late, when performed after the 10th day of mechanical ventilation. Tracheotomies were performed by two experienced ICU physicians at the bedside using the Ciaglia percutaneous technique (PT) with continuous bronchoscopic surveillance, or with an open surgical approach (OST) by two experienced ENT surgeons. The decision to perform tracheotomy was made by the treating physicians and was based on the predicted length of mechanical ventilation beyond 14 days post-intubation, and stable respiratory parameters (decreasing values of PEEP and FiO2, with plateau pressure less than 30 cm H_2_O, driving pressure less than 15 cm H_2_O, respiratory rate less than 35 breaths per minute with blood pH >7.25). Demographic and clinical data of the patients was collected, including age, sex, comorbidities, laboratory parameters. Two scales assessing the severity of critical illness were used: APACHE II (Acute Physiology and Chronic Health Evaluation consisting of physiological parameters, history of chronic illnesses and age) and SOFA (Sequential Organ Failure Assessment evaluating respiratory, cardiovascular, hepatic, coagulation, renal and neurological systems) scores [11, 12]. The ventilatory and gasometric parameters from the day of the tracheotomy and 48 hours after were also noted as well as data regarding complications and personnel infection with SARS-CoV-2 due to participation in the tracheotomy.

Data analysis was carried out using R: A Language and Environment for Statistical Computing, version 4.0.5. Data are presented as *n* (% of group) for nominal variables and as mean ± SD or median (Q1;Q3) for continuous data. Normality of distribution was assessed via Shapiro-Wilk test, based on visual assessment of histograms and based on the level of skewness and kurtosis. Comparison of groups for nominal data was based on chi-square test or Fisher exact test, as appropriate. Between-group analysis of continuous data was conducted using independent t test and Mann–Whitney *U* test, as appropriate. For groups comparison OR (odds ratio) for nominal variables and MD (mean/median difference) for continuous variables were calculated, both with 95% confidence interval. Additional analysis included logistic regression to determine factors significantly impacting risk of death. Univariate models were created using as predictors variables with *p* < 0.25 in groups comparison analysis. All tests were based on 0.05 significance level.

## Results

Between the 10th of November, 2020, and 28th of February, 2021, a number of 66 patients with COVID-related pneumonia were admitted to the Poznań University of Medical Science and included in the study. Their mean (SD) age was 63 [11] years and among them 48 (73%) were male. Mean (SD) SOFA and APACHE II score on admission were 9.7 (3.3) and 20.8 (7.8), respectively. 63 (95%) patients had chronic medical disease with hypertension in 50 (76%) patients, diabetes in 22 (33%) patients, obesity in 11 (16.7%), and chronic kidney disease in 9 (13.7%) patients, being the commonest among them. 60 (91%) of the analysed patients required mechanical ventilation with the mean (SD) duration of mechanical ventilation of 12.8 (8.8) days. 32 patients underwent tracheotomy within first 14 days of hospitalization. Among the rest of 34 nontracheotomized patients, 6 (9 %) were treated with HFNO (high flow nasal oxygenation) with a mean duration of 6.6 days, 8 (12%) patients were successfully extubated and 20 (59%) patients died before the decision to perform tracheotomy. Demographic, laboratory and clinical data of the patients with and without tracheotomy are presented in Table 1.

**Table 1:** Comparison of patients with and without tracheotomy

	All (N = 66)	Tracheotomy (N = 32)	Non-tracheotomy (N = 34)	OR/MD (95 CI)	*p*
Sex, male, n (%)	48 (72.7)	25 (78.1)	23 (67.6)	1.69 (0.49;6.11)	0.497
Age, years, mean ± SD	63.09 ± 11.36	59.94 ± 11.06	66.06 ± 10.97	− 6.12 (− 11.54;− 0.70)	0.028
SOFA, mean ± SD	9.71 ± 3.29	9.63 ± 2.86	9.79 ± 3.70	− 0.17 (− 1.79; 1.45)	0.836
APACHE, mean ± SD	20.83 ± 7.83	19.81 ± 7.20	21.79 ± 8.38	− 1.98 (− 5.82;1.85)	0.306
LIS (lung injury score), median (Q1;Q3)	3.50 (3.00;3.80)	3.50 (3.35;3.80)	3.40 (2.10;3.80)	0.10 (− 0.01;0.80)	0.184
Hospitalization time, days, mean ± SD	13.29 ± 8.71	19.19 ± 8.07	7.74 ± 4.76	11.45 (8.15; 14.75)	< 0.001
Result of ICU stay, n (%)					
Survival	30 (45.5)	16 (50.0)	14 (41.2)	1.42 (0.48;4.23)	0.621
Mortality	36 (54.5)	16 (50.0)	20 (58.8)		
Mechanical ventilation, days, median (Q1;Q3)	9.00 (4.75;18.25)	19.00 (13.50;21.00)	5.00 (1.25;8.00)	14.00 (10.00;16.00)	< 0.001
VAP (ventilatory associated pneumonia), n (%)	34 (51.5)	24 (75.0)	10 (29.4)	6.95 (2.16;24.82)	< 0.001
Ferritin on admission, mean ± SD	1 291.11 ± 670.33	1 281.69 ± 658.42	1 300.84 ± 693.18	− 19.15 (− 359.99;321.70)	0.911
IL6 on admission, median (Q1;Q3)	91.00 (31.00;215.00)	91.00 (44.00;207.50)	94.00 (26.50;209.25)	− 3.00 (− 50.00;50.00)	0.762
D-dimer on admission, median (Q1;Q3)	5.15 (1.70;20.00)	4.99 (1.86;17.18)	5.70 (1.66;21.00)	− 0.72 (− 4.59;2.18)	0.808
LDH on admission, median (Q1;Q3)	634.00 (425.50;748.00)	684.00 (491.50;779.00)	560.50 (372.00;695.25)	123.50 (− 19.00;235.00)	0.083
CRP on admission, mean ± SD	124.44 ± 92.85	134.50 ± 108.36	114.69 ± 75.30	19.81 (− 26.70;66.32)	0.397
PCT on admission, median (Q1;Q3)	0.51 (0.21;1.38)	0.47 (0.21;1.42)	0.57 (0.21;1.38)	− 0.11 (− 0.35;0.27)	0.916

Both groups compared with Fisher exact test for chi-square test for nominal variables and with t test or Mann–Whitney U test for continuous variables. OR—odds ratio in the tracheotomy vs. nontracheotomy group, MD—mean/median difference (the tracheotomy group minus nontracheotomy group), both with 95% confidence interval (CI)

25 (78%) patients underwent an early tracheotomy and 7 (22%) patients had late tracheotomy. The median duration of mechanical ventilation before the tracheotomy in the early group was 8 days [IQR 6-10], compared to 11 days [IQR 11-12.5]. *p* < 0,001) in the late group. Patients in the late tracheotomy group had significantly higher body weight (112.86 ± 23.9 kg vs. 87.3 ± 19.6 kg; *p* 0.031) and BMI (36.6 ± 9.3 vs. 28.1 ± 5.5 with a *p* value of 0.055 being almost statistically significant). There was no statistically significant difference in the PEEP value, FiO2 and P/F ratio either on the day of tracheotomy or 48 h after the tracheotomy in the early and late tracheotomy groups. However, a higher percentage of patients in the late tracheotomy group required controlled mode of ventilation 48 hours after the tracheotomy [3 (43%) vs. 2 (8%)]. A comparable number of patients was discharged from the ICU in both groups; 4 (57%) patients in the late group vs. 12 (48%) patients in the early group. Based on logistic regression, late tracheotomy was significantly impacted by weight and BMI. All factors were increasing the risk of late tracheotomy, for weight OR = 1.06 CI_95_[1.02; 1.12]; *p* = 0.021, for BMI OR = 1.20 CI_95_[1.04; 1.46]; p = 0.011.

Demographic and clinical parameters of the early and late tracheotomy patients are presented in Table 2.

**Table 2:** Tracheotomy patients—early vs. late tracheotomy

	Tracheotomy (N = 32)	Early (N = 25)	Late (N = 7)	OR/MD (95 CI)	*p*
Sex, male, n (%)	25 (78.1)	19 (76.0)	6 (85.7)	1.86 (0.16; 101.42)	> 0.999
Age, years, mean ± SD	59.94 ± 11.06	59.76 ± 11.81	60.57 ± 8.56	0.63 (-7.83; 9.46)	0.843
Weight, kg	92.88 ± 22.92	87.28 ± 19.65	112.86 ± 23.95	19.98 (3.00; 48.15)	0.031
BMI	29.97 ± 7.29	28.13 ± 5.55	36.56 ± 9.31	6.59 (-0.23;17.09)	0.055
Number of days from intubation to tracheotomy, median (Q1;Q3)	9.00 (6.00;10.00)	8.00 (6.00;9.00)	11.00 (11.00;12.50)	2.00 (1.00;7.00)	< 0.001
Number of days from tracheotomy to ICU discharge	10.00 (8.00;13.00)	10.00 (8.00;13.00)	9.50 (7.50;14.75)	-0.50 (-6.00;14.00)	0.807
Number of days from tracheotomy to death	9.00 (4.00;11.00)	9.50 (3.25;11.00)	7.50 (6.25;15.00)	-2.00 (-7.00;25.00)	> 0.999
SOFA, mean ± SD	9.63 ± 2.86	9.28 ± 2.94	10.86 ± 2.34	1.23 (-0.74;3.89)	0.164
APACHE, mean ± SD	19.81 ± 7.20	19.52 ± 7.53	20.86 ± 6.28	1.04 (-4.83;7.50)	0.644
LIS (lung injury score), median (Q1;Q3)	3.50 (3.35;3.80)	3.50 (3.30;3.65)	3.80 (3.65;3.80)	0.30 (0.01;0.50)	0.039
Hospitalization time, days, median (Q1;Q3)	19.00 (14.50;21.50)	18.00 (12.00;23.00)	19.00 (18.50;20.50)	1.00 (-3.00;9.00)	0.465
Result of hospitalization, n (%)					
Survival	16 (50.0)	12 (48.0)	4 (57.1)	0.70 (0.08;5.12)	> 0.999
Mortality	16 (50.0)	13 (52.0)	3 (42.9)		
Mechanical ventilation, days, median (Q1;Q3)	19.00 (13.50;21.00)	18.00 (12.00;21.00)	19.00 (18.50;20.50)	1.00 (-2.00;10.00)	0.375
VAP (ventilator-associated pneumonia) n (%)	24 (75.0)	19 (76.0)	5 (71.4)	0.80 (0.09;10.41)	> 0.999

Both groups compared with Fisher exact test for chi-square test for nominal variables and with t test or Mann–Whitney U test for continuous variables. OR—odds ratio in the late vs. early group, MD—mean/median difference (the late group minus early group), both with 95% confidence interval (CI)

During the analysed period, none out of 60 healthcare workers who participated in the tracheotomy procedure got infected with SARS-CoV-2.

Four patients (12.5%) underwent an open surgical tracheotomy while the rest 28 (87.5%)—a percutaneous tracheotomy. The overall rate of adverse events was 17%. Half [2] of the patients suffered from severe surgical site infection after the surgical tracheotomy and malpositioning of the tube requiring surgical handling, while in the percutaneous group only one minor stomal infection occurred and was treated with antibiotics alone. Bleeding occurred in 4 patients after percutaneous tracheotomy, all of them required blood products transfusion (3 of them were fully heparinized due to ECMO circuits).

An analysis comparing 32 patients with tracheotomies revealed 14 (44%) patients who survived and were discharged from the ICU and 18 (56%) patients who died—the mortality rate of patients who underwent tracheotomy was 56%. There was no statistically significant difference in the age, gender and BMI between the groups of survivors and non-survivors. The mean values in the SOFA and APACHE score on admission were comparable between the survivors and non-survivors (SOFA 9.7 ± 3.5 vs. 9.6 ± 1.9; *p* = 0.99 and APACHE 21.5 ± 7.6 vs. 17.6 ± 6.1; *p* = 0.12). Patients who survived had lower values of PEEP, FiO2 on the day of tracheotomy and 48 h after the procedure. The data presenting the main ventilation parameters are presented in Table 3.

**Table 3:** Tracheotomy patients—survivors vs. non-survivors

	Tracheotomy (N = 32)	Non-survivors (N = 18)	Survivors (N = 14)	OR/MD (95 CI)	*p*
PEEP on the day of tracheotomy	10.14 ± 2.71	11.29 ± 2.57	8.50 ± 2.02	2.79 (1.04;4.55)	0.003
FiO_2_ on the day of tracheotomy	40.17 ± 7.51	42.65 ± 8.31	36.67 ± 4.50	5.98 (1.05;10.91)	0.019
P/F on the day of tracheotomy	189.69 ± 52.93	176.59 ± 59.62	208.25 ± 36.33	− 31.66 (− 68.34;5.02)	0.088
PEEP 48 h after tracheotomy	9.04 ± 3.71	10.31 ± 3.77	7.33 ± 2.96	2.98 (0.36;5.60)	0.027
FiO2 48 h after tracheotomy	43.21 ± 12.15	50.06 ± 11.58	34.08 ± 4.48	15.98 (9.37;22.58)	< 0.001
P/F 48 h after tracheotomy	197.15 ± 68.72	152.17 ± 42.71	235.71 ± 64.55	− 83.55 (− 150.00;− 17.10)	0.019
Hospitalization time, days, median (Q1;Q3)	19.00 (14.50;21.50)	19.00 (12.75;20.75)	18.50 (17.00;22.50)	0.50 (− 3.00;8.00)	0.493
Mechanical ventilation, days, median (Q1;Q3)	19.00 (13.50;21.00)	19.00 (12.75;20.75)	18.50 (17.00;22.50)	0.50 (− 3.00;6.00)	0.595
VAP, n (%)	24 (75.0)	15 (83.3)	9 (64.3)	2.69 (0.41;21.67)	0.252

Both groups compared with Fisher exact test for chi-square test for nominal variables and with t test or Mann–Whitney *U* test for continuous variables. OR—odds ratio in the late vs. early group, MD—mean/median difference (the dead group minus survival group), both with 95% confidence interval (CI)

As per logistic regression analysis (Table 4), risk of death in tracheotomy patients was significantly growing with growing level of PEEP and FiO2 at the moment of decision on tracheotomy, OR = 1.91 CI_95_[1.23; 3.57]; p = 0.014 and OR = 1.18 CI_95_[1.03; 1.43]; *p* = 0.048 respectively. FiO2 level 48 h after tracheotomy was also significantly increasing the risk of death, OR = 1.29 CI_95_[1.11; 1.65]; *p* = 0.009.

**Table 4:** Logistic regression for death in tracheotomy patients

	OR	95% CI for OR	*p*
PEEP in the time of tracheotomy	1.912	1.233 to 3.568	0.014
FiO2 in the time of tracheotomy	1.178	1.026 to 1.433	0.048
P/F in the time of tracheotomy	0.987	0.970 to 1.002	0.121
PEEP 48 h after tracheotomy	1.350	1.040 to 1.974	0.056
FiO2 48 h after tracheotomy	1.290	1.110 to 1.648	0.009
P/F 48 h after tracheotomy	0.965	0.910 to 0.994	0.095
APACHE	1.087	0.979 to 1.231	0.140
LIS	1.690	0.891 to 2504.459	0.190
Pneumonia	3.667	0.814 to 20.503	0.106

OR—odds ratios for univariate logistic regression models (outcome variable: late tracheotomy  =  1, early tracheotomy  =  0) with 95% confidence interval (CI). Predictors included all variables with *p* < 0.25 in group comparison

## Discussion

### Technique

The majority (88%) of cases in our study underwent percutaneous tracheotomy and surgical method was preferred when patients were very obese and with difficult access to the anterior surface of the neck. The proportion of these two techniques differed considerably between previous studies on tracheotomy in COVID-19 patients [13]. Basing on recent meta-analysis [14], the open tracheotomy was performed in 2047 patients (55.7%), and percutaneous tracheotomy in 990 patients (43.4%) with a hybrid technique in 23 patients.

### Complications

Regarding the type of tracheotomy, percutaneous or surgical in patients with COVID-19, there were no significant differences in complication rates (bleeding and stomal infections) between the two methods according to Long et al**.** [15]. The authors reported complications in 16% of procedures—3% of stomal infections only in the open tracheotomy group, minor bleeding in 7.5 % and a need to perform an open procedure after tube dislodgement during percutaneous technique in one of 144 patients.

Similar results were observed in study conducted by Rovira et al. [16] where complications occurred in 18.9% of 201 patients with no differences between an open and percutaneous technique, both during (bleeding, hypoxia, misplacement, tracheal injury) and after the procedure (bleeding, cuff leak, tube dislodgement, hypoxia and pneumothorax). Breik et al. [17] reported 13% of complications with self-limiting bleeding and tube dislodgement the commonest among them, with similar rates in percutaneous and surgical technique. In Tang et al. study [18] bleeding occurred in 17.5%, (5% required blood products transfusion), tracheotomy infection in 1.2% and subcutaneous and mediastinal emphysema in 1.2% of patients with no difference between the early (< within 14 days following intubation) and late tracheotomy group. We observed a similar complication rate of 17%. Due to the small number of patients who underwent surgical tracheotomy and different anatomical characteristics of patients, we cannot compare these two techniques in our study.

### Survival

In our study, the survival rate was similar in patients with and without tracheotomy (50% vs. 41%, respectively). Patients with tracheotomy had much longer mean mechanical ventilation time (19 vs. 5 days) and had higher incidence of ventilator associated pneumonia (75% vs. 29.4 %). However, the group of nontracheotomized patients included two extremes—there were patients whose clinical status improved fast and allowed extubation within 7 days, or were without secured airways on HFNO and, on the other hand, there were deteriorating patients with a poor prognosis due to severity of gas exchange abnormalities and multiorgan failure. In the study by Long et al. [15], including 144 patients, the timing of performing tracheotomy was 3 weeks and the mortality rate on the 26th post-tracheotomy day was 7.5%. The authors conclude that the surprisingly low mortality rate was due to the fact that a great part of the severely ill patients were likely to die before the consideration for tracheotomy. They emphasize the relatively prolonged time of weaning from mechanical ventilation because of several weeks of sedation, muscle paralysis (aimed to enable lung protective ventilation and to suppress exaggerated respiratory drive to prevent self-inflicted lung injury) and slow improvement in lung function due to COVID-19 infection which is similar with our observations that the group that underwent tracheotomy had much longer time of mechanical ventilation and length of stay as well as VAP occurrence.

### Viral transmission

The other issue concerning safety of tracheotomy in COVID patients is the safety of the medical personnel involved in the procedure. Postponing the tracheotomy beyond the 14th and even 21st day after intubation was based on the opinion that after such a long time the viral shedding was diminished and the risk of infection with the SARS-CoV-2 virus to the medical staff during this aerosol generating procedure was minimized [8, 9, 19, 20]. Some institutions recommended delaying tracheotomy until COVID-19 testing was negative [13]. All medical staff in our institution were wearing appropriate personal protective equipment, including face shields, FFP 3 masks, double gloves and surgical gowns. We did not observe coronavirus infection in health care personnel performing the procedure, which is consistent with the experiences in other centers regardless of the tracheotomy timing [21, 22]. In meta-analysis [23] on safety of tracheotomy in COVID-19 patients, three out of the included 58 studies reported small number of health care professionals who were infected with SARS-CoV-2 virus (one case each in 2 studies and 7.7% in the third, with a rate of 11% in health care workers not involved in tracheotomy procedures).

### Timing

Prolonged orotracheal ventilation necessitates deeper sedation or even muscle paralysis leading to increased risk of ICU acquired weakness, prolonged mechanical ventilation and hospital length of stay as well as poses a risk of tracheal stenosis [24]. Timing of tracheotomy in critically ill patients is still inconclusive, with a definition of early tracheotomy varying from 4 to 14 days among the studies. A Cochrane review [25] showed lower mortality rates and greater probability of discharge from ICU in the early (less than 10 days postintubation) tracheotomy group with inconsistent data regarding the time of mechanical ventilation and pneumonia occurrence. On the other hand, TracMan trial [26] found no difference in mortality or duration of mechanical ventilation in the early (within 4 days following intubation) tracheotomy group. In our retrospective study, all tracheostomies, except one case, were performed within 14 days from intubation, with a mean time of 9 days. In a meta-analysis including tracheotomies in 462 COVID-19 patients [23], the pooled cumulative incidence of early tracheotomy (within 7 days from intubation) was 5.2%, the pooled cumulative incidence of intermediate tracheotomy (between day 8 and 13) was 21.2%, and the pooled cumulative incidence of late tracheotomy (14 days or more after intubation) was 71.5%. The estimated overall mean timing was calculated as 13.6 ± 3.1 days after intubation, with a range of 0–42 days. Tang et al. [18] found out that tracheotomy before the 14th day was associated with increased mortality rate, but patients in the late tracheotomy group had lower SOFA and APACHE II scores. Botti et al. emphasize due to lack of establishment of optimal timing that tracheostomy should be performed on case-by-case basis and based on local healthcare resources and potential benefits for patients [27].

However, during the pandemic, the approach to timing of tracheotomy changed significantly. Chao et al. [19], who originally recommended postponing the performance of tracheotomy with an open technique beyond 21 days from intubation, changed their management during the course of SARS-CoV-2 pandemic. An updated practice from the authors’ institution showed mean timing of tracheotomy between the 10th and 14th day, and the percutaneous technique being performed as a standard [19, 21]. Similarly, despite the New York Head and Neck Society standard to perform tracheotomy on the 14th day, their mean timing was 10 days from intubation [28]. A study from Brazil showed that COVID patients with severe comorbidities have improved prognosis with an early tracheotomy performed 4–5 days from intubation [21]. Rosano et al. [22] analysed a group of 121 COVID patients with a median of tracheotomy performed on the 6th day and 98% of cases performed before the 10th day of intubation. In a multivariable analysis, early percutaneous tracheotomy was independently associated with decreased hospital mortality. 55 % of tracheotomized patients were discharged from the hospital.

We performed a comparison analysis of patients with an early tracheotomy performed in 25 patients (median time of 8 days postintubation) and late tracheotomy in 7 patients (median 11 days). Only one patient underwent tracheotomy more than 14 days after implementing invasive mechanical ventilation. There were no differences in both groups in terms of mortality or ventilator parameters on the day of tracheotomy. The decision to perform tracheotomy was based on relative stability of lung mechanics and gas exchange and those patients with higher PEEP and FiO2 values, as well as those who still benefited from prone position were deferred from the procedure until reaching a stable P/F ratio.

There were no differences between the early and late tracheotomy group in terms of duration of mechanical ventilation (18 vs. 19 days), ICU length of stay (18 vs. 19 days) and VAP occurrence (76 vs. 71.4%). The cumulative time of mechanical ventilation was longer due to transfer of the patients to other facilities and rehabilitation centers with a mean time to decannulation averaging 42 days. A recent study by Liao et al. [29], on a group of 1000 ICU tracheotomized patients, revealed a decannulation rate of 16.7%, with an average time to decannulation of 40.9 days, which is consistent with our findings on COVID-19 patients with ARDS.

In a Spanish national cohort study, including 1890 COVID-19 patients who underwent tracheotomy, the authors found that 1 month after the performance of the tracheotomy, 52% of the patients were weaned from mechanical ventilation, 35% still required mechanical ventilation and 24% died [13]. As mentioned above, there are several benefits of an early tracheotomy with shorter duration of analgosedation, mechanical ventilation, ICU and hospital stay, but are they always true for the weaning process of COVID-19 pneumonia patients is still an open question.

The authors of meta-analysis [14] found that tracheotomy led to successful mechanical ventilation weaning in 54.9% of patients, decannulation in 34.9% of cases within 18 days on average, and postulate that there is a need for finding prognostic factors for successful outcome.

## Prognostic factors

Sustained FiO2 ≤ 50% and PEEP ≤ 8 cm H_2_O any time during the course of treatment are strong predictive factors for a good outcome, raising the potential for these patients to be weaned down early, thus increasing ICU capacity [30]. No demographic or laboratory data as well as severity of illness based on prognostic scales utilized in intensive care unit did not predict the successful weaning and decannulation. In our study, an analysis comparing 32 patients who underwent tracheotomy showed that the survivors had lower PEEP, FiO2 and better P/F ratio on the day of tracheotomy and 48 hours post-tracheotomy. In a logistic regression model, PEEP and FiO2 on the day of tracheotomy and 48 hours post-tracheotomy were independent factors increasing risk of dying. It was proven in this study that those with lower PEEP and FiO2 values were extubated earlier, those with lower parameters in the time of tracheotomy and 48 hours after the procedure had a higher overall survival rate.

## Limitations

The biggest limitation of this study is the small sample size—among 66 patients, 32 were tracheotomized and among them 25 underwent the procedure before 10 days and 7 after 10 days postintubation.

## Conclusion

As suggested in our study early percutaneous tracheotomy is safe (both in terms of risk of viral transmission to healthcare personnel and complication rate) and feasible in COVID-19 patients. Stability of gas exchange and ventilatory parameters are the main prognostic factors of the outcome and when they are achieved tracheostomy may be safely performed.

## References

[1] Griffiths MJD, McAuley DF, Perkins GD, Barret N, Blackwood B, Boyle A (2019). Guidelines on the management of acute respiratory distress syndrome. BMJ Open Resp Res.

[2] Papazian L, Aubron C (2019). Formal guidelines: management of acute respiratory distress syndrome. Ann Intensive Care.

[3] Bosel J, Schiller P, Hook Y, Andes M, Neumann JO, Poli S (2013). Stroke-related early tracheostomy versus prolonged orotracheal intubation in neurocritical care trial (SETPOINT): a randomized pilot trial. Stroke..

[4] Robba C, Galimberti S, Graziano F, Wiegers E, Lingsma HF, Iaquaniello C (2020). Tracheostomy practice and timing in traumatic brain-injured patients: a CENTER-TBI study. Intensive Care Med.

[5] Hosokawa K, Nishimura M, Egi M (2015). Timing of tracheotomy in ICU patients: a systematic review of randomised controlled trials. Crit Care.

[6] Sommer DD, Engels PT, Weitzel EK (2020). Recommendations form the CSO-HNS taskforce on performance of tracheotomy during the COVID-19 pandemic. J Otolaryngol Head Neck Surg.

[7] McGrath BA, Ashby N, Birchall M (2020). Multidisciplinary guidance for safe tracheostomy care during COVID 19 pandemic: the NHS national patient safety improvement programme (NatPatSIP). Anaesthesia.

[8] American Academy of Otolaryngology and Head and Neck Surgery (2020) AAO position statement: tracheotomy recommendations during the COVID-19 pandemic. https://www.entnet.org/resource/tracheotomy-recommendations-during-the-covid-19-pandemic-2/. Accessed 27 Apr 2021

[9] Miles BA, Schiff B, Ganly I, Ow T, Cohen E, Genden E (2020). Tracheostomy during SARS-CoV-2 pandemic: recommendations from the New York Head and Neck Society. Head Neck.

[10] Schultz MJ, Pattnaik R, Dondorp AM (2020). Walking the line between benefit and harm from tracheostomy in COVID-19. Lancet Respir Med.

[11] Knaus WA, Draper EA, Wagner DP, Zimmerman JE (1985). APACHE II: a severity of disease classification system. Crit Care Med.

[12] Vincent JL, Moreno R, Takala J, Willatts S, De Mendonça A, Bruining H (1996). The SOFA (Sepsis-related Organ Failure Assessment) score to describe organ dysfunction/failure. On behalf of the working group on sepsis-related problems of the European Society of Intensive Care Medicine. Intensive Care Med..

[13] Bier-Laning C, Cramer JD, Roy S, Palmieri PA, Amin A, Anon JM (2021). Tracheostomy during the COVID-19 pandemic: comparison of international perioperative care protocols and practices in 26 countries. Otolaryngol Head Neck Surg.

[14] Benito DA, Bestourous BE, Tong JY (2021). Tracheotomy in COVID-19 patients: a systematic review and meta-analysis of weaning, decannulation and survival. Otolaryngol Head Neck Surg.

[15] Long SM (2021). Percutaneous and open tracheostomy in patients with COVID-19: comparison and outcomes of an institutional series in New York City. Ann Surg.

[16] Rovira A, Tricklebank S, Surda P (2021). Open versus percutaneous tracheostomy in COVID-19: a multicentre comparison and recommendation for future resource utilisation. Eur Arch Otorhinolaryngol.

[17] Queen Elizabeth Hospital Birmingham COVID-19 Airway Team (2020). Safety and 30-day outcomes of tracheostomy for COVID-19: a prospective observational cohort study. BJA.

[18] Tang Y, Wu Y, Zhu F, Yang X, Huang C, Hou G, Xu W (2020). Tracheostomy in 80 COVID-19 patients: a multicenter, retrospective observational study. Front Med.

[19] Chao TN, Harbison SP, Braslow BM (2020). Outcomes after tracheostomy in COVID-19 patients. Ann Surg.

[20] Sommer DD, Engels PT, Weitzel EK, Khalili S, Corsten M, Tewfik MA, Fung K, Cote D, Gupta M, Sne N, Brown TFE, Paul J, Kost KM, Witterick IJ (2020). Recommendations from the CSO-HNS taskforce on performance of tracheotomy during the COVID-19 pandemic. J Otolaryngol Head Neck Surg.

[21] Schultz MJ, Teng MS, Brenner MJ (2020). Timing of tracheostomy for patients with COVID 19 in the ICU—setting precedent in unprecedented times. JAMA Otolaryngol Head Neck Surg.

[22] Rosano A, Martinelli E, Fusina F, Albani F, Caserta R, Morandi A (2021). Early percutaneous tracheostomy in coronavirus disease 2019: association with hospital mortality and factors associated with removal of tracheostomy tube at ICU discharge. A cohort study on 121 patients. Crit Care Med.

[23] Staibano P, Levin M, McHugh T (2021). Association of tracheostomy with outcomes in patients with COVID-19 and SARS-CoV-2 transmission among health care professionals: a systematic review and meta-analysis. JAMA Otorlaryngol Head neck Surg.

[24] Piazza C, Filuaro M, Dikkers FG (2021). Long-term intubation and high rate of tracheostomy in COVID-19 patients might determine an unprecedented increase in airway stenosis: a call to action from European Laryngological Society. Eur Arch Otorhinolaryng.

[25] Andriolo BNG, Andriolo RB, Saconato H (2015). Early versus late tracheostomy for critically ill patients. Cochrane Database Syst Rev.

[26] Young D, Harrison DA, Cuthbertson BH (2013). Effect of early vs late tracheostomy placement on survival in patients receiving mechanical ventilation: the TracMan randomized trial. JAMA.

[27] Botti C, Lusetti F, Peroni S, Neri T, Castellucci A, Salsi P, Ghidini A (2021). The role of tracheotomy and timing of weaning and decannulation in patients affected by severe COVID-19. Ear Nose Throat J.

[28] Angel L, Kon ZN, Chang SH (2020). Novel percutaneous tracheostomy for critically ill patients with COVID-19. Ann Thorac Surg.

[29] Liao DZ, Mehta V, Kinkhabwala CM, Li D, Palsen S, Schiff BA (2020). The safety and efficacy of open bedside tracheotomy: a retrospective analysis of 1000 patients. Laryngoscope.

[30] Stubington TJ, Mallick AS, Garas G, Stubington E, Reddy C, Mansuri MS (2020). Tracheotomy in COVID-19 patients: optimizing patient selection and identifying prognostic indicators. Head Neck.

